# 2,3,7,8-Tetrachlorodibenzo-*p*-Dioxin (TCDD)-Inducible Poly-ADP-Ribose Polymerase (TIPARP/PARP7) Catalytic Mutant Mice (*Tiparp^H532A^*) Exhibit Increased Sensitivity to TCDD-Induced Hepatotoxicity and Lethality

**DOI:** 10.1093/toxsci/kfab075

**Published:** 2021-06-15

**Authors:** David Hutin, Alexandra S Long, Kim Sugamori, Peng Shao, Sachin Kumar Singh, Marit Rasmussen, Ninni Elise Olafsen, Solveig Pettersen, Giulia Grimaldi, Denis M Grant, Jason Matthews

**Affiliations:** 1 Department of Pharmacology and Toxicology, University of Toronto, Toronto, M5S 1A8 Ontario, Canada; 2 Department of Immunology, Oslo University Hospital, 0372 Oslo, Norway; 3 Department of Nutrition, Institute of Basic Medical Sciences, University of Oslo, 0372 Oslo, Norway

**Keywords:** aryl hydrocarbon receptor, wasting syndrome, ADP-ribosylation, 2,3,7,8-tetrachlorodibenzo-p-dioxin, TCDD-inducible poly-ADP-ribose polymerase (TIPARP), poly-ADP-ribose polymerase 7 (PARP7), ADP-ribosyltransferase diphtheria toxin-like 14 (ARTD14)

## Abstract

2,3,7,8-tetrachlorodibenzo-*p*-dioxin (TCDD)-inducible poly-adenosine diphosphate (ADP)-ribose polymerase (TIPARP/PARP7), an aryl hydrocarbon receptor (AHR) target gene and mono-ADP-ribosyltransferase, acts as part of a negative feedback loop to repress AHR signaling. This process is prevented by a single H532A mutation in TIPARP that destroys its catalytic activity. We hypothesized that the loss of TIPARP catalytic activity would increase sensitivity to TCDD-induced toxicity *in vivo*. To test this, we created a catalytically deficient mouse line (*Tiparp^H532A^*) by introducing a single H532A mutation in TIPARP. Treatment of mouse embryonic fibroblasts or hepatocytes isolated from *Tiparp^H532A^* mice confirmed the increased TCDD-induced expression of the AHR target genes *Cyp1a1*, *Cyp1b1*, and *Tiparp*. *Tiparp^H532A^* mice given a single injection of 10 µg/kg TCDD, a nonlethal dose in *Tiparp^+/+^* mice, did not survive beyond day 10. All *Tiparp^+/+^* mice survived the 30-day treatment. TCDD-treated *Tiparp^H532A^* mice displayed increased expression of AHR target genes, increased steatohepatitis and hepatotoxicity. Hepatic RNA-sequencing revealed 7-fold more differentially expressed genes in *Tiparp^H532A^* mice than in *Tiparp^+/+^* mice (4542 vs 647 genes) 6 days after TCDD treatment. Differentially expressed genes included genes involved in xenobiotic metabolism, lipid homeostasis and inflammation. Taken together, these data further support TIPARP as a critical negative regulator of AHR activity and show that loss of its catalytic activity is sufficient to increase sensitivity to TCDD-induced steatohepatitis and lethality. Since TIPARP inhibition has recently emerged as a potential anticancer therapy, the impact on AHR signaling, TCDD and polycyclic aromatic hydrocarbon toxicity will need to be carefully considered under conditions of therapeutic TIPARP inhibition.

##  

2,3,7,8-Tetrachlorodibenzo-*p*-dioxin (TCDD)-induced poly-adenosine diphosphate (ADP)-polymerase (TIPARP), also known as poly-ADP-ribose polymerase 7 (PARP7) and ADP-ribosyltransferase diphtheria toxin-like 14 (ARTD14), is a mono-ADP-ribosyltransferase and a member of the PARP family. The PARP family consists of 17 members that regulate many cellular signaling pathways, including metabolism, DNA repair, protein stability, gene regulation, inflammation, and immunity (Gibson and Kraus, 2012). PARPs function by transferring ADP-ribose (ADPr) from nicotinamide adenine dinucleotide (NAD^+^) onto themselves and other protein substrates, releasing nicotinamide. The majority of PARPs transfer a single molecule of ADPr (mono-ADP-ribosylation; MARylation), rather than several ADPr moieties (poly-ADP-ribosylation; PARylation), onto their substrates (Hottiger *et al.*, 2010; Krishnakumar and Kraus, 2010; Vyas *et al.*, 2014). PARPs are activated in response to internal and external cellular stresses, such as viral infection, genotoxicity, reactive oxygen species, and chemical toxicity (Cohen and Chang, 2018; Gibson and Kraus, 2012; Grunewald *et al.*, 2020; Kraus and Hottiger, 2013). However, in contrast to other well-established posttranscriptional modifications like phosphorylation, methylation, acetylation and ubiquitination, the effect of MARylation on protein function is not well understood (Cohen and Chang, 2018).

Multiple studies indicate that TIPARP plays important roles in the regulation of gene expression, innate immunity, interferon signaling, and cancer (MacPherson *et al.*, 2013; Palavalli Parsons *et al.*, 2021; Yamada *et al.*, 2016; Zhang *et al.*, 2020). TIPARP is regulated by platelet derived growth factor (Chen *et al.*, 2004), nuclear receptor family members (Bindesboll *et al.*, 2016; Kamata *et al.*, 2021), hypoxia-inducible factor 1α (HIF-1α, Zhang *et al.*, 2020), viral infection (Yamada *et al.*, 2016), including Severe acute respiratory syndrome coronavirus 2 (SARS-CoV-2) (Heer *et al.*, 2020), and the aryl hydrocarbon receptor (AHR, Ma *et al.*, 2001; MacPherson *et al.*, 2013). TIPARP MARylates AHR and represses AHR signaling through a negative feedback loop that depends on its catalytic activity (Gomez *et al.*, 2018; MacPherson *et al.*, 2013).

AHR is a ligand activated transcription factor mostly studied due to its ability to mediate the toxicity of numerous chemicals and environmental contaminants including TCDD. AHR is now recognized to be an essential gatekeeper that integrates dietary, environmental, and endogenous ligand signals to modulate immune cell homeostasis, inflammation and cancer (Safe *et al.*, 2017). Although the definitive endogenous AHR ligand has not been identified, 6-formylindolo[3,2-*b*]carbazole (FICZ) and kynurenine (KYN) are potential candidates (Opitz *et al.*, 2011; Wincent *et al.*, 2009). In the canonical AHR signaling pathway, ligand binding to cytosolic AHR causes its translocation to the nucleus and its binding to specific DNA sequence elements (termed aryl hydrocarbon response elements or dioxin response elements) together with its heterodimerization partner, AHR nuclear translocator. AHR regulates the expression of many genes, including cytochrome P450 1A1 (*CYP1A1*), *CYP1B1*, AHR repressor (*AHRR*), and *TIPARP* (Ma *et al.*, 2001; Whitlock, 1999; Zhang *et al.*, 1998). TCDD causes diverse toxic outcomes, including immunosuppression, steatohepatitis, impaired reproduction and a lethal wasting syndrome (Pohjanvirta and Tuomisto, 1994; Poland and Knutson, 1982). TCDD-induced lethality varies widely among species and within rodent strains, with lethality from a single dose of TCDD ranging from 1 µg/kg in guinea pigs to >5000 µg/kg in hamsters (Pohjanvirta and Tuomisto, 1994; Poland and Knutson, 1982). For most mouse strains, lethality occurs 3 weeks after a single dose of 200–500 µg/kg of TCDD (Pohjanvirta, 2009; Pohjanvirta and Tuomisto, 1994). The molecular mechanisms of TCDD-induced wasting syndrome and the vast differential species sensitivity to TCDD remain unclear. However, an intact AHR signaling pathway is absolutely required for TCDD-induced wasting syndrome (Fernandez-Salguero *et al.*, 1996). For example, transgenic mice overexpressing AHR have enhanced toxicity to TCDD, while *Ahr*^*−*^^*/*^^*−*^ mice are resistant to the effects of TCDD (Fernandez-Salguero *et al.*, 1996; Lee *et al.*, 2010; Walisser *et al.*, 2005).

We recently reported that whole body and hepatocyte specific knockout of *Tiparp*, an AHR repressor, leads to increased AHR activity and increased sensitivity to TCDD-induced toxicities, including steatohepatitis, hepatotoxicity and lethal wasting syndrome (Ahmed *et al.*, 2015; Hutin *et al.*, 2018). Since AHR is MARylated by TIPARP and the ability of TIPARP to repress AHR is dependent on its catalytic activity (Gomez *et al.*, 2018; MacPherson *et al.*, 2013), we hypothesized that loss of TIPARP catalytic activity would be sufficient to increase AHR activity and sensitivity to TCDD toxicities. Here, we describe the generation of *Tiparp^H532A^* catalytic mutant mice by clustered regularly interspaced short palindromic repeats (CRISPR)/CRISPR associated protein (Cas9) gene editing. These mice were used to further investigate the role of TIPARP in AHR signaling and TCDD-induced toxicity. Our data show that loss of TIPARP’s catalytic activity alone increases the sensitivity of mice to TCDD-induced steatohepatitis and lethality. Pharmacological TIPARP inhibition has emerged as a potential anticancer therapy, mainly due to TIPARP’s role in interferon signaling. However, based on the data presented here, the impact on AHR signaling and TCDD toxicity will need to be carefully considered during therapeutic TIPARP inhibition.

## MATERIALS AND METHODS

###  

####  

##### Chemicals

Dimethyl sulfoxide (DMSO), FICZ (>95% purity) and KYN (>97.5% purity) were purchased from Sigma-Aldrich (St Louis, Missouri). TCDD (>99% purity) was purchased from Accustandard (New Haven, Connecticut). Ribon-2397 (RBN-2397; >99% purity) was purchased from MedChemExpress (Monmouth Junction, New Jersey). Corn oil (CO; 100% purity) was purchased from a local grocer. The Infinity alanine aminotransferase (ALT) Liquid Stable Reagent was purchased from Thermo Fisher Scientific (Middletown, Virginia) for use in the determination of ALT activity. All other chemicals were purchased from Sigma-Aldrich unless stated otherwise.

##### Generation of *Tiparp^H532A^* mice

*Tiparp^H532A^* mice were created by Cyagen (Santa Clara, California) using a guide RNA (gRNA) sequence targeting the amino acid residue H532 located in exon 6 of *Tiparp* and a donor targeting sequence, flanked by 60 bp of homologous sequence containing the H532A (CAT to GCC) mutation. To create the mutation, Cas9 mRNA, single guide RNA (sgRNA), and oligo donor were coinjected into zygotes from C57BL/6 mice for knockin *Tiparp^H532A^* mouse production. The founder F0 female homozygous C57BL/6 mouse was crossed with C57BL/6 WT and the F1 pups were genotyped by PCR, followed by sequence analysis and *Hpa*II restriction analysis, which was introduced by the CAT to GCC mutation. A total of 2 male and 4 female F1 heterozygous mice were received from Cyagen by the Division of Comparative Medicine at the University of Toronto (Toronto, Canada). The colony, which is congenic C57BL/6, was expanded and maintained by breeding *Tiparp*^+/H532A^ heterozygous mice. Genotyping was done with the REDExtract-N-Amp Tissue PCR Kit (Sigma-Aldrich). The PCR primers used were Fwd: 5′-CAACACAGTAAACACTTGCATAGA-3′; Rev: 5′-ATGCCATGACTGCCCATTGTGTAT-3′. The PCR product was purified with GenepHlow Gel/PCR isolation kit (FroggaBio, Concord, Canada) and digested with *Hpa*II (Thermo Fisher Scientific) overnight at 37°C. The *WT* allele generates a 530 bp product, whereas the *H532A* allele generates 320 bp and 210 bp products.

##### *In vivo* TCDD treatment studies

In all experiments, 8- to 10-week-old male mice were used. For the 6 hour study, *Tiparp^+/+^* or *Tiparp^H532A^* mice were treated with a single intraperitoneal (i.p.) injection of 10 µg/kg TCDD, and livers were excised and flash frozen 6 h later. The 10 µg/kg dose of TCDD was dissolved in a mixture of CO:DMSO (90:10, referred to as CO), whereas the 100 µg/kg dose of TCDD was dissolved in DMSO as described previously (Ahmed *et al.*, 2015; Hutin *et al.*, 2018). For the subacute TCDD toxicity studies, mice were treated with a single i.p. injection of 10 µg/kg of TCDD and sacrificed on day 6. For the survival studies, mice were followed for up to 30 days after a single injection of 10 or 100 µg/kg of TCDD. Control mice received equivalent weight-adjusted volumes of CO. The time point for euthanasia was determined based on endpoint criteria for our study: a loss of 20% body weight or other indications of acute distress. All control mice were euthanized to match the endpoints of TCDD-sensitive mice. Whole blood was obtained from the saphenous vein for serum ALT analysis as described previously (Ahmed *et al.*, 2015). Liver, thymus, and white adipose tissue (WAT) were dissected and weighed. Livers from *Tiparp^H532A^* mice treated with vehicle or 10 µg/kg TCDD were collected either on day 6 or on the day of euthanasia in the survival studies. Care and treatment of animals followed the guidelines set by the Canadian Council on Animal Care, and all protocols were approved by the University of Toronto Animal Care Committee.

##### Derivation of mouse embryonic fibroblasts

*Tiparp^+/+^* and *Tiparp^H532A^* fibroblasts were prepared from E14.5 embryos derived from mating heterozygous mice as described previously (MacPherson *et al.*, 2013). Primary fibroblasts from *Tiparp^+/+^* and *Tiparp^H532A^* siblings were immortalized at passage 2 after transfection with Simian virus large T antigen (SV40) in pSG5 (Merck, Oakville, Canada) and a puromycin resistance plasmid, and selected in puromycin-containing medium. The genotypes of the mouse embryonic fibroblasts (MEFs) were verified by PCR.

##### Generation of anti-TIPARP antibody

The antibody was generated as described previously (Rasmussen *et al.*, 2021). Briefly, recombinant 6× histidine-tagged murine TIPARP 1-320 was expressed and purified from *Escherichia* *coli* strain BL-21. The recombinant protein (50 µg in RIBI adjuvant, Millipore Sigma, Oakville, Ontario) was injected into 8-week-old female BALB/C mice at 2-week intervals, followed by 2 injections of 20 µg protein in RIBI adjuvant. Hybridomas were generated and those producing specific antibodies that recognize murine TIPARP were selected by ELISA screening.

##### RNA extraction and gene expression analysis

Tissues were removed, washed in ice-cold PBS, weighed, and flash frozen in liquid nitrogen. Frozen livers were homogenized in TRIzol reagent (Thermo Fisher Scientific). Total RNA was isolated using the Aurum RNA isolation kit (BioRad, Hercules, California) and reverse transcribed as previously described (Ahmed *et al.*, 2015). Hepatocytes were isolated and cultured as previously described (Hutin *et al.*, 2018). Briefly, livers from *Tiparp^+/+^* or *Tiparp^H532A^* male mice (8–10 weeks old) were perfused with liver perfusion medium (Invitrogen) for 10 min, followed by liver digestion medium for 10 min. Hepatocytes were seeded at a final density of 2.5 × 10^5^ cells/well in type I collagen coated 12-well plates in attachment medium (William’s E media, 10% dextran-coated charcoal [DCC] stripped fetal bovine serum [FBS], 1× penicillin/streptomycin [PEST], and 10 nM insulin). The medium was exchanged 2 h after plating, and experiments were performed the following day. Ligands were added to the cells in M199 media with 5% DCC-FBS and cells were harvested 6 h after ligand treatment for RNA extraction. MEFs were seeded at a final density of 1.0 × 10^5^ cells/well in 12-well plates in Dulbecco’s Modified Eagle Medium (DMEM) containing 10% FBS, PEST, and L-glutamine. The following morning, cells were treated with ligands and harvested after 6 h for RNA extraction. First strand synthesis and real time-quantitative PCR (RT-qPCR) were done as described previously (Ahmed *et al.*, 2015; Hutin *et al.*, 2018). Primers used to amplify target transcripts are described in Supplementary Table 1 or elsewhere (Ahmed *et al.*, 2015). All genes were normalized to TATA binding protein expression levels and analyzed using the comparative *C*_T_ (ΔΔ *C*_T_) method.

##### RNA-sequencing and data analysis

Global gene expression analysis using RNA-sequencing was carried out by the Norwegian High Throughput Sequencing Centre (Department of Medical Genetics, Oslo, Norway). Total RNA was isolated with the total RNA isolation kit according to the manufacturer’s protocol (RNeasy Mini Kit, Qiagen). The quality of total RNA was evaluated using the Agilent 2100 Bioanalyzer (Agilent, Palo Alto, California) with the RNA 6000 Nano LabChip kit. RNA-seq libraries were prepared with Illumina TruSeq RNA Library Prep Kit v2 and sequenced using Illumina HiSeq2500 to obtain 150-bp paired-end reads. The sequencing depth for each sample was >20 million reads. The reads were aligned with TopHat to mm10, UCSC database with default parameters. Assembly of transcripts and differential gene expression analysis were performed using Features Counts and DESeq2 (Love *et al.*, 2014). Differentially expressed genes were defined as those with an adjusted *p* value < .05 AND an absolute linear fold-change value of ≥ 2. Pathway analysis was carried out using Ingenuity Pathway Analysis version 60467501. RNA-seq datasets were deposited in the Gene Expression Omnibus (accession number GSE167205).

##### Ethoxyresorufin-O-deethylase activity assay

MEFs were plated at a density of 10 000 cells per well in 96-well plates. The following day, the medium was changed, and the cells were treated with DMSO or increasing concentrations of TCDD for 24 h. The following morning, the media was aspirated and replaced with 100 µl of a Tris-sucrose buffer (Tris 50 mM, sucrose 0.1 M, pH 8.0) containing 8 µM 7-ethoxyresorufin and 10 µM dicoumarol. The plates were incubated at 37°C for 45 min after which time 75 µl of ice-cold methanol was added to each well and plates were mixed at 300 rpm for 2 min. Ethoxyresorufin-O-deethylase (EROD) activity was measured by fluorescence spectrometry using an excitation wavelength of 540 nm and emission wavelength of 590 nm. The protein concentrations in each well were determined by adding 100 µl of bicinchoninic acid assay reagent.

##### Histology

Hematoxylin and eosin and oil red O/hematoxylin staining were performed following standard methods (Hutin *et al.*, 2018) with representative images provided. For picrosirius red staining, paraffin-embedded liver tissues were sectioned and stained for the presence of collagen using standard picrosirius red staining techniques. Paraformaldehyde-fixed, Optimal cutting temperature (OCT)-embedded or paraffin-embedded tissues were sent to the Histology Core Facility at the Princess Margaret Cancer Centre, (Toronto, Canada) for all histology sample processing, staining, and scanning of stained slides.

##### ADP-ribosylation assay

COS-1 cells were seeded in 6-well plates (2.0 × 10^5^ cells/well) in DMEM medium containing 10% FBS and 1% PEST. The next morning, cells were transfected with murine TIPARP in the form of GFP-TIPARP or GFP-TIPARP^H532A^ using Lipofectamine 2000. Twenty-four hours later, cells were washed with ice-cold PBS and lysed in cell lysis buffer (200 mM NaCl, 1% NP40 and 20 mM HEPES at pH 7.4, and supplemented with 1× protease inhibitor cocktail [Sigma-Aldrich]). Lysates were clarified by centrifugation and incubated with anti-GFP (Life Technologies, Carlsbad, California; 3E6). Protein complexes were captured with protein G Dynabeads (Life Technologies) after a 2 h incubation at 4°C. Beads were washed 4 times with cell lysis buffer and eluted in 1× sample buffer. Samples were separated by SDS-Polyacrylamide gel electrophoresis (PAGE) and transferred to PVDF membranes. Membranes were probed with anti-poly/mono-ADPr (Cell Signaling Technology, Danvers, Massachusetts; E6F6A), or anti-GFP (JL8; Takara Bio, Mountain View, California) followed by incubation with the appropriate secondary antibodies.

##### Western blotting

For TIPARP protein detection, whole cell extracts of MEFs were obtained by lysing cells in RIPA lysis buffer. Twenty micrograms of total protein were separated by SDS-PAGE and transferred to a PVDF membrane. Membranes were blocked for 1 h at room temperature in 5% non-fat milk dissolved in Tris buffered saline (TBS)-0.1% Tween20 (blotto buffer). Membranes were then incubated overnight at 4°C with anti-TIPARP antibodies from clones 2G9 and 1E9, which were pooled at 1:1000 dilution in blotto buffer, 1:1000 dilution of anti-PARP7 (Abcam 84664; lot no. GR3304056-5) or 1:10 000 dilution of anti-AHR (Enzo Life Sciences SA210; lot no. 04011942) followed by incubation with the appropriate secondary antibodies. For detection of hepatic AHR, approximately 50–80 mg frozen liver tissue was homogenized in 400 µl RIPA lysis buffer. The homogenate was sonicated with Bioruptor on the low setting at 4°C for 5 min, 30 s on and 30 s off. After rotating for 15 min at 4°C, the samples were centrifuged at 20 000 × g for 10 min at 4°C. Ten micrograms of total protein were separated by SDS-PAGE and transferred to a PVDF membrane. Membranes were incubated overnight at 4°C with 1:10 000 dilution of anti-AHR (Enzo Life Sciences SA210; lot no. 05011813) followed by incubation with the appropriate secondary antibody. PVDF membranes were stripped and incubated with anti-β-actin antibody 1:4000 (Sigma-Aldrich; A-2228).

##### Tissue lipid analysis

Livers from mice treated with 10 µg/kg TCDD were flash frozen and used to measure tissue triglyceride levels using an ethanolic saponification method as described previously (Jouihan, 2012; Norris *et al.*, 2003). Briefly, 70–75 mg of frozen tissue was incubated overnight in ethanolic KOH (2:1 ethanol: 30% potassium hydroxide) at 55°C to lyse the tissue and hydrolyze the triglycerides. The amount of free glycerol in the liver samples and serial dilutions of glycerol (Millipore Sigma; G7793) were determined by coupled enzyme reactions initiated by addition of free glycerol reagent (Millipore Sigma; F6428) with absorbance measured at 540 nm. Liver triglyceride content (mg triglyceride/g liver) was calculated as cuvette triolein equivalent mg/dl/liver tissue weight (g).

##### Statistical analysis

Unless otherwise stated, all data were presented as means with SEM. Two-way analysis of variance (ANOVA) followed by Sidak’s post hoc or 1-way ANOVA followed by a Tukey’s post hoc tests were used to assess statistical significance (*p *<* *.05) using GraphPad Prism 8 Software (San Diego, California) or R version 3.4.1.

## RESULTS

###  

#### Generation of Conditional Tiparp^H532A^ Mice

**Figure 1.**
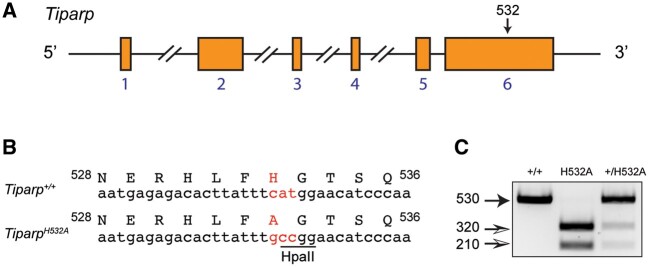
Generation of *Tiparp^H532A^* catalytic mutant mice using CRISPR/Cas9-mediated gene editing. A, Schematic diagram of *Tiparp* wildtype allele with exons, and the approximate location of H532, which was targeted to create a *Tiparp^H532A^* mutant mouse. Exons are indicated by the orange boxes and their numbers are indicated in blue font. B, To simplify genotyping, an *Hpa*II restriction enzyme site was introduced simultaneously with the replacement of histidine 532 with alanine. C, Representative genotyping of wildtype, H532A mutant, and heterozygous mice.

The *Tiparp^H532A^* mice used in this study were generated by CRISPR/Cas9 gene editing by Cyagen. The H532A (CAT to GCC) mutation was introduced into exon 6 of *Tiparp* by homology-directed repair (Figure 1A). The change in genomic sequence resulted in the introduction of a *Hpa*II restriction enzyme site (Figure 1B). The pups were genotyped by PCR, followed by sequence analysis and *Hpa*II restriction analysis (Figure 1C). Six heterozygous (*Tiparp^+/H532A^*) F1 mice (2 males and 4 females) were shipped to the Department of Comparative Medicine at the University of Toronto. The *Tiparp^+/H532A^* mice were backcrossed for 6 generations onto C57BL/6J mice (Jackson Labs), before expanding the colony and initiating the studies.

#### Loss of TIPARP Catalytic Activity Increases AHR Signaling In Vitro and In Vivo

**Figure 2.**
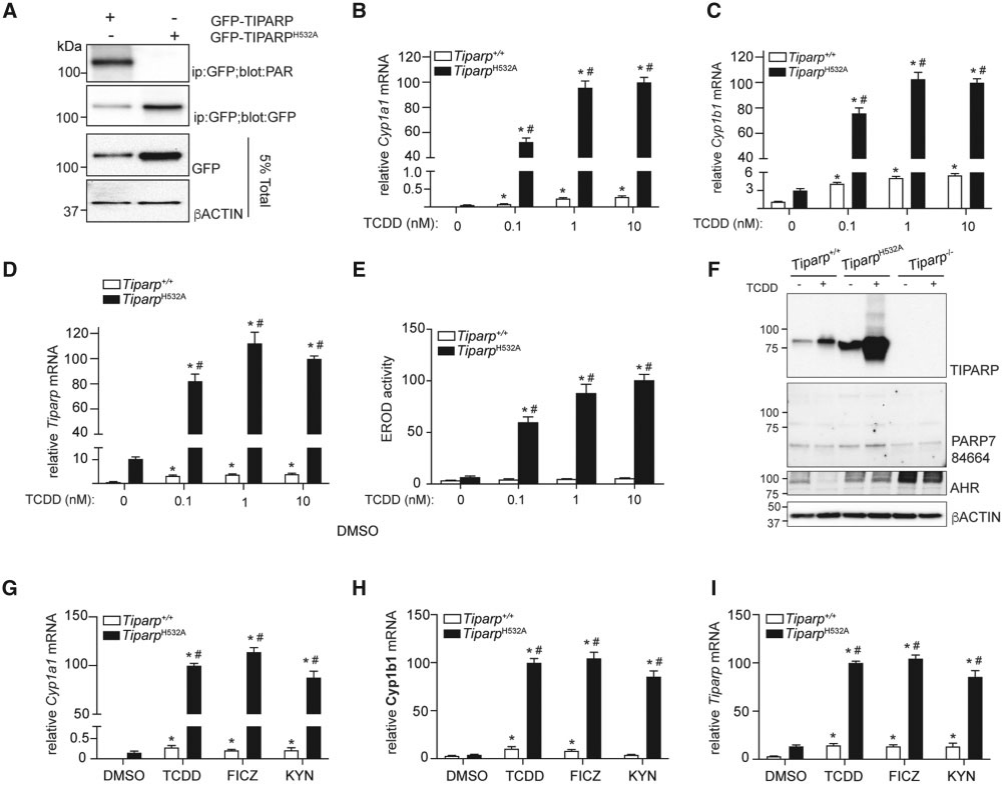
Increased aryl hydrocarbon receptor (AHR) signaling in MEFs isolated from *Tiparp^H532A^* compared with *Tiparp^+/+^* mice. A, *In vitro* adenosine diphosphate (ADP)-ribosylation assay. GFP-**2,3,7,8-**tetrachlorodibenzo-*p*-dioxin (TCDD)-inducible poly-adenosine diphosphate (ADP)-ribose polymerase (TIPARP) wildtype, but not GFP-TIPARP^H532A^ exhibits ADP-ribosyltransferase activity in extracts isolated from transfected COS-1 cells. Increased TCDD-induced mRNA expression levels of (B) *Cyp1a1*, (C) *Cyp1b1*, and (D) *Tiparp* in treated immortalized mouse embryonic fibroblasts (MEFs) isolated from *Tiparp^H532A^* and *Tiparp^+/+^* mice. E, Increased ethoxyresorufin-O-deethylase activity in MEFs isolated from *Tiparp^+/+^* and *Tiparp^H532A^* mice. F, 2,3,7,8-tetrachlorodibenzo-*p*-dioxin (TCDD) increases TIPARP protein expression in MEFs. MEFs were treated with 10 nM TCDD for 4 h. The membrane was blotted with our lab generated anti-TIPARP antibody, a commercially available anti-PARP7 (Abcam; ab84664) antibody or anti-AHR (Enzo; SA210). Anti-PARP7 (ab84664) did not detect endogenous TIPARP. Increased mRNA expression levels of (G) *Cyp1a1*, (H) *Cyp1b1*, and (I) *Tiparp* in *Tiparp^+/+^* and *Tiparp^H532A^* mice treated with 10 nM TCDD, 10 nM 6-formylindolo[3,2-*b*]carbazole (FICZ) or 200 µM kynurenine (KYN) for 6 h. All real time-quantitative PCR data are shown relative 100% to 10 nM TCDD from *Tiparp^H532A^* MEFs for each gene. *p <* *.05 2-way analysis of variance (ANOVA) followed by Sidak’s post hoc test compared with genotyped-matched control-treated MEFs. ^#^*p *<* *.05 2-way ANOVA followed by Sidak’s post hoc test compared with time-matched TCDD-treated *Tiparp^+/+^* MEFs. Figure depicts the mean ± SEM (*n *=* *3).

To verify that the H532A mutation resulted in a catalytically deficient murine TIPARP, we compared the auto-mono-ADP-ribosylation activity of GFP-TIPARP wildtype and GFP-TIPARP^H532A^. Immunoprecipitated GFP-TPARP was probed with the anti-PAR antibody that detects ADP-ribosylated proteins. Mono-ADP-ribosylation was detected on GFP-TIPARP, but no ADP-ribosylation was detected on the catalytic mutant, GFP-TIPARP^H532A^ (Figure 2A). Similar to that observed for human TIPARP, introduction of the H532A mutation into murine TIPARP increased its protein stability compared with wildtype protein (MacPherson *et al.*, 2013).

Consistent with TIPARP’s role as a negative regulator of AHR activity, exposure of MEFs isolated from *Tiparp^H532A^* mice to TCDD for 6 h resulted in increased mRNA levels of *Cyp1a1* and *Cyp1b1*, as well as increased EROD activity after 20 h compared with similarly treated MEFs derived from *Tiparp^+/+^* mice (Figs. 2B–E). *Tiparp* mRNA levels were also increased in TCDD-treated *Tiparp^H532A^* mice compared with *Tiparp^+/+^* mice. This was expected since *Tiparp*, like *Cyp1a1* and *Cyp1b1*, is an AHR target gene. Unlike *Tiparp*^*−*^^*/*^^*−*^ mice, the introduction of the H532A mutation into the *Tiparp* gene would still result in a full-length mRNA with a the change H532A in the translated protein. Protein expression analysis using an in-house generated mouse monoclonal IgG2a anti-TIPARP antibody confirmed the TCDD-dependent increase in TIPARP protein levels in treated *Tiparp^+/+^* and *Tiparp^H532A^* MEFs (Figure 2F). In support of the data in Figure 2A, TIPARP^H532A^ protein was expressed at a much higher level than wildtype protein in both the vehicle and TCDD-treated samples. TIPARP protein was not detected in extracts isolated from *Tiparp*^*−*^^*/*^^*−*^ MEFs. However, a commercially available anti-TIPARP/PARP7 antibody failed to detect the increase in TIPARP^H532A^ protein levels. Constitutive AHR protein levels were similar in *Tiparp^+/+^* and *Tiparp^H532A^* MEFs, but increased in *Tiparp*^*−*^^*/*^^*−*^ MEFs (Figure 2F). However, the *Tiparp^+/+^* MEFs were generated from breeding *Tiparp^+/H532A^* mice and are not the matched wild-type for the *Tiparp*^*−*^^*/*^^*−*^ MEFs, which might contribute to the observed differences. For *Tiparp^H532A^* and *Tiparp*^*−*^^*/*^^*−*^ MEFs, we observed reduced TCDD-induced AHR degradation compared with *Tiparp^+/+^* MEFs. This may contribute to the observed increased AHR signaling and is in agreement with a previous report (Ahmed *et al.*, 2015). Six hour treatment with FICZ or KYN, 2 endogenous AHR agonists, also increased *Cyp1a1*, *Cyp1b1*, and *Tiparp* expression levels in *Tiparp^H532A^* compared with *Tiparp^+/+^* MEFs, showing that the increased AHR responsiveness was not limited to TCDD as a ligand (Figs. 2G–I).

**Figure 3.**
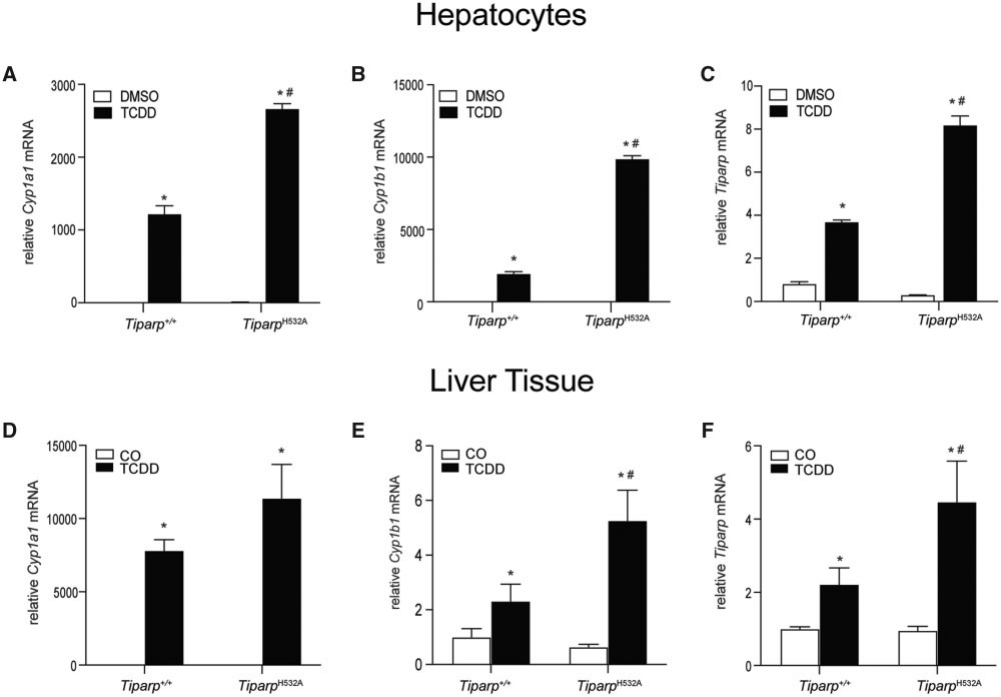
Loss of 2,3,7,8-tetrachlorodibenzo-*p*-dioxin (TCDD)-inducible poly-adenosine diphosphate-ribose polymerase (TIPARP) catalytic activity increased TCDD-induced aryl hydrocarbon receptor signaling in hepatocytes and hepatic tissue. Increased mRNA expression levels of (A) *Cyp1a1*, (B) *Cyp1b1*, and (C) *Tiparp* in hepatocytes isolated from *Tiparp^+/+^* and *Tiparp^H532A^* mice treated with 1 nM TCDD for 6 h. Increased hepatic mRNA expression levels of (D) *Cyp1a1*, (E) *Cyp1b1*, and (F) *Tiparp* in *Tiparp^+/+^* and *Tiparp^H532A^* mice treated for 6 h with 10 µg/kg TCDD. *p <* *.05 2-way analysis of variance (ANOVA) followed by Sidak’s post hoc test compared with genotyped-matched control-treated mice. ^#^*p *<* *.05 2-way ANOVA followed by Sidak’s post hoc test compared with time-matched TCDD-treated *Tiparp^+/+^* mice. Figure depicts the mean ± SEM (*n *=* *4). Corn oil (CO).

We next examined whether loss of TIPARP catalytic activity affected AHR target gene expression in hepatocytes and liver tissue isolated from *Tiparp^+/+^* and *Tiparp^H532A^* mice 6 h after treatment with 10 µg/kg TCDD. TCDD treatment caused a significant increase in *Cyp1a1*, *Cyp1b1*, and *Tiparp* mRNA levels in *Tiparp^H532A^* hepatocytes compared with similarly treated hepatocytes from *Tiparp^+/+^* mice (Figs. 3A–C). TCDD treatment caused a significant increase in the expression of *Cyp1a1*, above that observed in vehicle controls, but due to interanimal variability, no significant differences were observed between genotypes (Figure 3D). Significant increases in *Cyp1b1* and *Tiparp* mRNA levels were observed in *Tiparp^H532A^* mice compared with *Tiparp^+/+^* mice (Figs. 3E and 3F).

#### Tiparp^H532A^ Mice Exhibit Increased Sensitivity to TCDD-Induced Toxicity and Lethality

**Figure 4.**
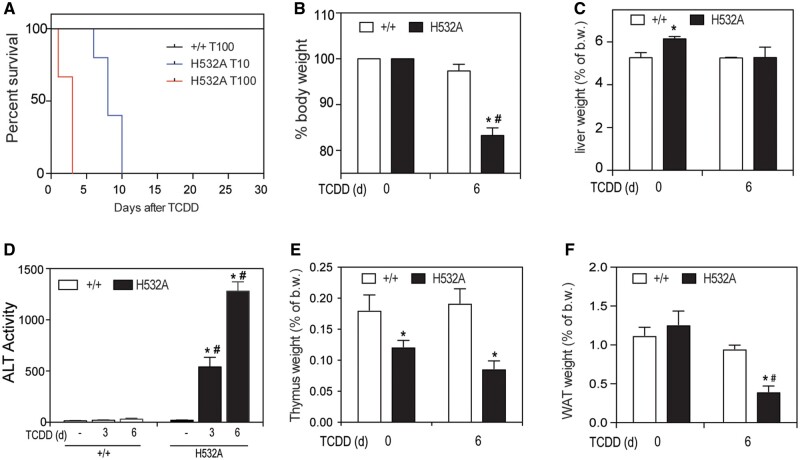
Increased sensitivity of *Tiparp^H532A^* mice to 2,3,7,8-tetrachlorodibenzo-*p*-dioxin (TCDD)-induced hepatotoxicity and lethality. A, Kaplan-Meier survival curves for *Tiparp^+/+^* treated with 100 µg/kg, and *Tiparp^H532A^* mice treated with a single injection of 10 (in blue) or 100 µg/kg (in red) TCDD (*n *=* *4–6). B, % body weight relative to pretreatment (C) liver somatic index, (D) serum alanine aminotransferase activity, (E) thymus somatic index, and (F) epididymal white adipose tissue somatic index at the days (d) indicated (*n *=* *4–6). *p <* *.05 2-way analysis of variance (ANOVA) followed by Sidak’s post hoc test compared with genotyped-matched control-treated mice. ^#^*p *<* *.05 2-way ANOVA followed by Sidak’s post hoc test compared with time-matched TCDD-treated *Tiparp^+/+^* mice. Figure depicts the mean ± SEM.

To determine the sensitivity of *Tiparp^H532A^* mice to TCDD-induced toxicity, *Tiparp^H532A^* and *Tiparp^+/+^* mice were given a single i.p. injection of 10 or 100 µg/kg TCDD and monitored for up to 30 days, as previously described (Ahmed *et al.*, 2015). All *Tiparp^+/+^* mice were normal in physical appearance at the end of the 30-day observation period, whereas no TCDD-treated *Tiparp^H532A^* mice survived the 30-day study (Figure 4A). *Tiparp^H532A^* mice treated with 100 µg/kg TCDD became weakened and moribund and were humanely euthanized between days 2 and 3, while those treated with 10 µg/kg TCDD were euthanized between days 7 and 10. *Tiparp^H532A^* mice treated with 10 µg/kg TCDD had significant body weight loss by 6 days after treatment (Figure 4B). Increased liver weights were observed in TCDD-treated *Tiparp^+/+^* mice, but not in *Tiparp^H532A^* mice (Figure 4C). Serum ALT activity, a marker of hepatotoxicity, was significantly increased in TCDD-treated *Tiparp^H532A^* mice as early as day 3, whereas no increase above controls was observed in *Tiparp^+/+^* mice (Figure 4D). Thymic involution, a well-known endpoint associated with TCDD toxicity, was observed in both genotypes at day 6 (Figure 4E). Similar to our previous report of TCDD-treated *Tiparp*^*−*^^*/*^^*−*^ mice, a significant decrease in epididymal WAT weight was observed in *Tiparp^H532A^* mice, but not in *Tiparp^+/+^* mice (Figure 4F). These data support the importance of TIPARP in regulating AHR action and show that loss of its catalytic activity in mice increases their sensitivity to TCDD toxicity.

#### Increased AHR-Regulated Gene Expression and Hepatotoxicity in Tiparp^H532A^ Mice After Treatment With 10 µg/kg TCDD

**Figure 5.**
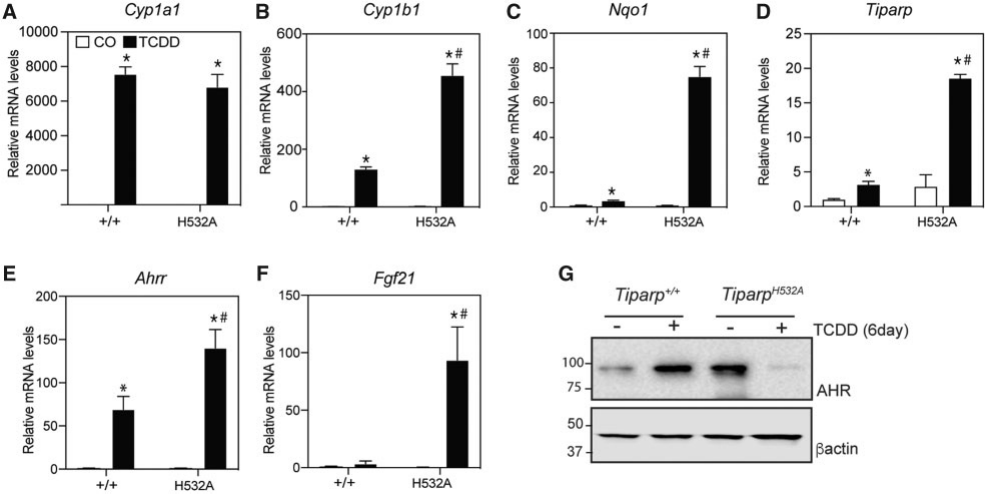
Increased hepatic gene expression of aryl hydrocarbon receptor (AHR)-regulated genes. Mice were treated with a single injection of 10 µg/kg 2,3,7,8-tetrachlorodibenzo-*p*-dioxin (TCDD), or corn oil (CO) (vehicle) alone, and euthanized 6 days later. RNA and real time-quantitative PCR for (A) *Cyp1a1*, (B) *Cyp1b1*, (C) *Nqo1*, (D) *Tiparp*, (E) *Ahrr*, and (F) *Fgf21* were performed as described in the materials and methods section (*n *=* *5). G, Representative western blot (*n *=* *3) of hepatic AHR protein levels in *Tiparp^+/+^* and *Tiparp^H532A^* mice 6 days after treatment with 10 µg/kg TCDD or CO (vehicle). *p <* *.05 2-way ANOVA followed by Sidak’s post hoc test compared with genotyped-matched control-treated mice. ^#^*p *<* *.05 2-way ANOVA followed by Sidak’s post hoc test compared with time-matched TCDD-treated *Tiparp^+/+^* mice. Figure depicts the mean ± SEM.

*Tiparp^H532A^* mice treated with 10 µg/kg TCDD for 6 days exhibited increased mRNA expression levels of many AHR target genes including *Cyp1b1*, *Nqo1*, *Ahrr*, *Tiparp*, and *Fgf21* compared with similarly treated *Tiparp^+/+^* mice (Figs. 5A–F). Although there was a trend for increased *Cyp1a1* mRNA levels in treated *Tiparp^H532A^* compared with *Tiparp^+/+^* mice, this difference did not reach statistical significance (Figure 5A). Constitutive hepatic AHR protein levels were elevated in liver extracts from *Tiparp^H532A^* mice compared with those from *Tiparp^+/+^* mice (Figure 5G). AHR levels were increased 6 days after treatment with 10 µg/kg TCDD. Despite elevated hepatic AHR gene expression levels, very low levels of hepatic AHR were detected in *Tiparp^H532A^* mice. However, the high level of hepatotoxicity might have impacted AHR protein levels in TCDD-treated *Tiparp^H532A^* mice compared with comparably treated *Tiparp^+/+^* mice.

**Figure 6.**
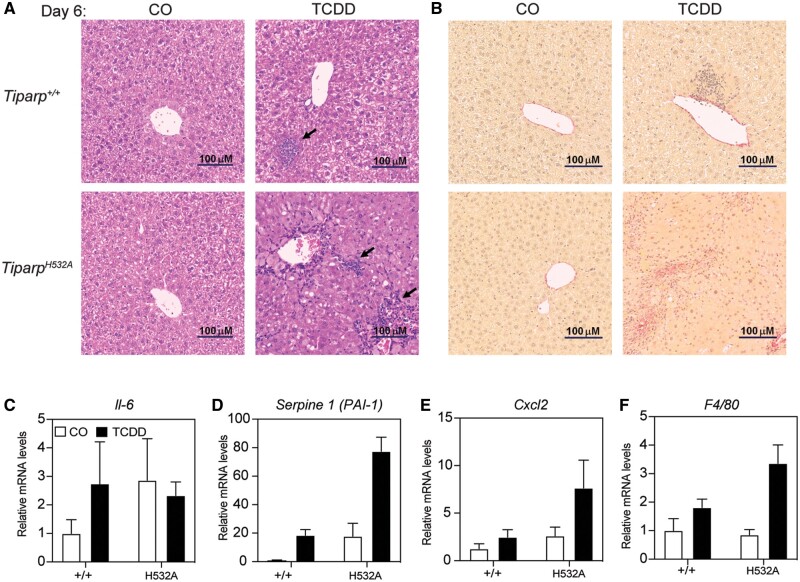
Increased hepatic inflammation in 2,3,7,8-tetrachlorodibenzo-*p*-dioxin (TCDD)-treated *Tiparp^H532A^* compared with *Tiparp^+/+^* mice. A, Representative hematoxylin and eosin staining of livers from *Tiparp^+/+^* and *Tiparp^H532A^* mice (*n *=* *4). The arrows indicate focal inflammatory infiltration. B, Representative picrosirius red staining of livers from *Tiparp^+/+^* and *Tiparp^H532A^* mice (*n *=* *4). Control animals were injected with CO and were euthanized on day 6. All images are to the same scale. Hepatic gene expression levels of (C) *interleukin 6*, (D) *Serpine 1*, (E) *Cxcl2*, and (F) *F4/80* were analyzed as described in the methods. *p <* *.05 2-way analysis of variance (ANOVA) followed by Sidak’s post hoc test compared with genotyped-matched control-treated mice. ^#^*p *<* *.05 2-way ANOVA followed by Sidak’s post hoc test compared with TCDD-treated *Tiparp^+/+^* mice. Figure depicts the mean ± SEM (*n *=* *4).

As an independent measure of liver toxicity, livers were sectioned and stained with hematoxylin and eosin. CO-treated *Tiparp^+/+^* and *Tiparp^H532A^* mice had histologically normal liver architecture (Figure 6A). On day 6, TCDD-treated *Tiparp^+/+^* livers exhibited slight hepatocyte cytoplasmic clearing within periportal regions and inflammatory cell infiltration (Figure 6A). In contrast, day 6 *Tiparp^H532A^* livers were characterized by inflammatory infiltration and a predominant microvesicular steatosis.

Sustained exposure to TCDD results in hepatic fibrosis in mice, which is dependent on AHR and is therefore not observed in *Ahr^−^^/^^−^* mice (Pierre *et al.*, 2014). To determine if a single dose of 10 µg/kg TCDD was sufficient to induce fibrosis in *Tiparp^H532A^* mice, liver sections were stained for the presence of collagen using picrosirius red. Livers from CO-treated mice and wildtype TCDD-treated mice sacrificed 6 days after treatment displayed collagen staining only within the vasculature (Figure 6B). In contrast, livers from TCDD-treated homozygous *Tiparp^H532A^* mice displayed increased picrosirius red staining that paralleled the bridging inflammatory infiltration, indicative of fibrotic changes (Figure 6B).

We next determined the mRNA levels of known AHR-regulated cytokines (Casado *et al.*, 2011; Matsubara *et al.*, 2012). TCDD-treatment did not affect hepatic interleukin 6 levels in either genotype (Figure 6C). However, TCDD-treated *Tiparp^H532A^* mice had increased hepatic expression of *Serpine 1* (also known as plasminogen activator inhibitor-1 [PAI-1]), chemokine (C-X-C motif) ligand 2 (*Cxcl2*) and *F4/80* when compared with *Tiparp^+/+^* mice (Figs. 6D and 6F). These data are consistent with increased hepatic inflammation in TCDD-treated *Tiparp^H532A^* compared with *Tiparp^+/+^* mice.

#### Loss of TIPARP Catalytic Activity Increases TCDD-Induced Steatohepatitis

**Figure 7.**
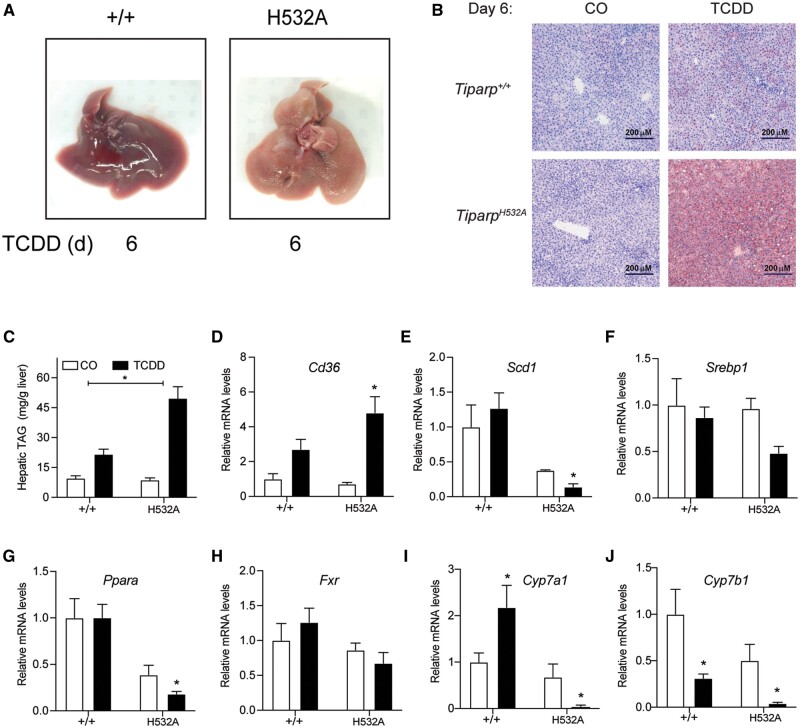
Increased steatohepatitis in 2,3,7,8-tetrachlorodibenzo-*p*-dioxin (TCDD)-treated *Tiparp^H532A^* compared with *Tiparp^+/+^* mice. A, Livers from male *Tiparp^+/+^* and *Tiparp^H532A^* mice given a single intraperitoneal injection of corn oil or 10 µg/kg TCDD and euthanized after 6 days (*n *=* *5). B, Oil Red O and hematoxylin stained liver sections from *Tiparp^+/+^* and *Tiparp^H532A^* mice. All images are to the same scale. C, Hepatic triacyl glyceride (TAG) levels were determined as described in the methods. Hepatic mRNA levels of (D) *Cd36*, (E) *Scd1*, (F) *Srebp1*, (G) *Ppara*, (H) *Fxr*, (I) *Cyp7a1*, and (J) *Cyp7b1* were determined as described in the methods. *p <* *.05 by 2-way analysis of variance followed by Sidak’s post hoc test compared with genotype-matched vehicle-treated mice. Figure depicts the mean ± SEM (*n *=* *4) Corn oil (CO); treatment day (d).

Livers from vehicle-treated *Tiparp^+/+^* and *Tiparp^H532A^* mice were macroscopically normal (Figure 7A). Livers from *Tiparp^+/+^* mice were enlarged and slightly pale in color 6 days after TCDD treatment (Figure 7A). Livers from TCDD-treated *Tiparp^H532A^* mice were pale in color at day 6, suggesting a high level of lipid accumulation. Livers from vehicle-exposed *Tiparp^+/+^* and *Tiparp^H532A^* mice were negative for the presence of neutral lipids as indicated by oil red O staining (Figure 7B). Small droplets of lipid were seen in the livers of *Tiparp^+/+^* mice on day 6 after TCDD treatment, while those from similarly treated *Tiparp^H532A^* mice had substantial intracytoplasmic lipid accumulation. In agreement with the oil red O staining, significantly higher hepatic triacyl glyceride levels were observed in TCDD-treated *Tiparp^H532A^* compared with *Tiparp^+/+^* mice (Figure 7C).

We then analyzed the hepatic levels of transcripts encoding genes involved in lipid uptake, lipogenesis, and cholesterol/bile acid metabolism. Consistent with previous studies (Ahmed *et al.*, 2015; Hutin *et al.*, 2018), the expression of lipid uptake transporter, scavenger receptor encoded by cluster of differentiation 36 (*Cd36)*, was higher in treated *Tiparp^H532A^* compared with wildtype mice (Figure 7D). Hepatic expression of lipogenic genes including stearoyl-CoA desaturase (*Scd1*), and sterol regulatory element-binding transcription factor 1 (*Srebp1*), were significantly decreased in TCDD-treated *Tiparp^H532A^* mice compared with *Tiparp^+/+^* mice (Figs. 7E and 7F). Peroxisome proliferator activating receptor α (*Ppara*; *Nr1c1*), farnesoid X receptor (*Fxr*; *Nr1h4*), as well as *Cyp7a1* and *Cyp7b1*, the rate limiting enzymes in bile acid synthesis, were also significantly decreased in *Tiparp^H532A^* compared with *Tiparp^+/+^* mice (Figs. 7G–J). These data suggest that the increased sensitivity of *Tiparp^H532A^* mice to TCDD-induced steatohepatitis is due to increased lipid uptake rather than increased hepatic lipogenesis.

#### Hepatic Gene Expression Analysis

**Figure 8.**
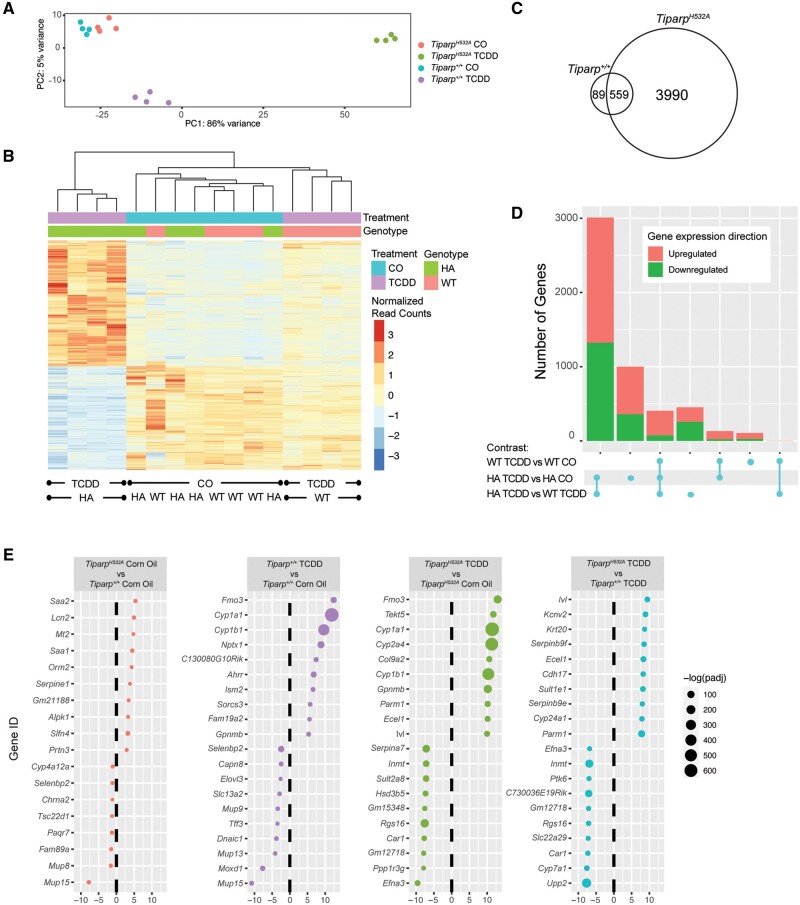
Hepatic gene expression profiling in TCDD-treated *Tiparp^H532A^* compared with *Tiparp^+/+^* mice. A, Principal component analysis of all samples. B, Heat map displaying the relative expression levels in each sample for all significant genes.^*a*^ Genes and individual samples are arranged by hierarchical clustering. Treatment group and genotype are indicated with colored bars at the top of the heatmap and represented as normalized read counts, or reads per kilobase of transcript per million reads mapped. C, Venn diagram of differentially regulated TCDD-induced hepatic genes in *Tiparp^+/+^* and *Tiparp^H532A^* mice treated for 6 days.^*b*^ D, An upset plot showing the number of differentially expressed genes^*b*^ in common between genotype or treatment group contrasts. On this graph, each row corresponds to a contrast, while each column corresponds to an intersection between contrasts. Circles connected by lines indicate the participation of the indicated contrasts in the intersection. E, The top 10 upregulated and top 10 downregulated differentially expressed genes,^*b*^ grouped by treatment or genotype contrast. Genes are listed by their MGI gene symbol and ranked by fold change.^*a*^For this analysis, significant genes are those that have an adjusted *p* value < .05 for the *Tiparp^H532A^* TCDD versus *Tiparp^H532A^* CO contrast. ^*b*^Genes are considered to be differentially expressed if they have an absolute linear fold change ≥ 2 and an adjusted *p* value < .05. Abbreviations: TCDD, 2,3,7,8-tetrachlorodibenzo-*p*-dioxin; CO, corn oil; WT, wildtype (*Tiparp^+/+^*); HA, H532A (*Tiparp^H532A^*).

**Figure 9.**
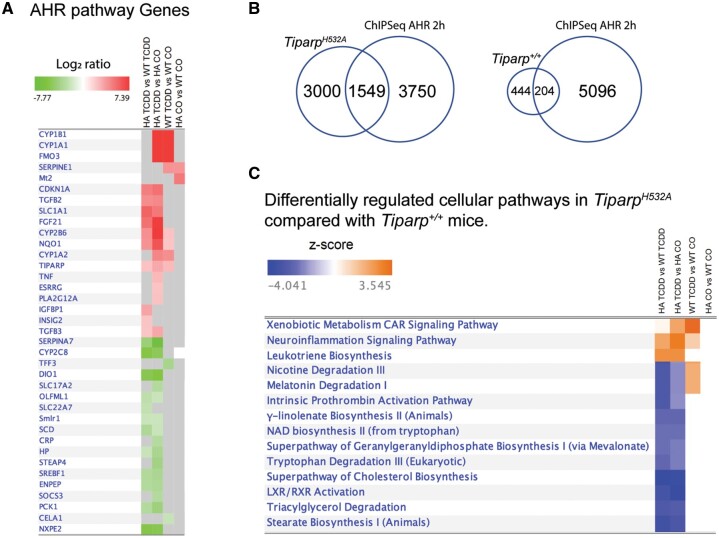
Aryl hydrocarbon receptor (AHR) gene battery and ingenuity pathway analysis (IPA) of hepatic gene expression changes in 2,3,7,8-tetrachlorodibenzo-*p*-dioxin (TCDD)-treated *Tiparp^H532A^* compared with *Tiparp^+/+^* mice. A, Heatmap of the significant differentially expressed genes^*a*^ for selected genes within the IPA AHR pathway, for all contrasts. Red represents upregulated genes and green represents downregulated genes. Gray squares represent genes that did not meet our critical adjusted *p* value cutoff (<.05). For the full gene list please see Supplementary Table 6. B, Venn diagram of the overlap in the number of hepatic genes changed in TCDD-treated *Tiparp^H532A^* or *Tiparp^+/+^* compared with TCDD-induced hepatic AHR-bound regions from (Dere *et al.*, 2011). C, Canonical pathway analysis of the differentially expressed genes^*a*^ for all contrasts. Filters for this analysis were set to z-score ≥ absolute 2.2 and Benjamini-Hochberg adjusted *p* value ≤ .05. Blue represents inhibited pathways and orange represents activated pathways. This figure was trimmed for presentation; for the full gene list please see Supplementary Table 7. ^*a*^Genes are considered to be differentially expressed if they have an absolute linear fold change ≥ 2 and an adjusted *p* value < .05.

To identify gene pathways that are uniquely regulated in *Tiparp^H532A^* mice and that might explain their increased sensitivity to TCDD-induced toxicity, we compared hepatic gene expression profiles 6 days after TCDD or CO treatment of *Tiparp^H532A^* and *Tiparp^+/+^* mice. Principal component analysis (PCA, Figure 8A) and hierarchical clustering of the genes in the individual samples showed distinct clustering of the TCDD-treated *Tiparp^H532A^* and *Tiparp^+/+^* animals, whereas the CO-treated samples clustered independently of genotype (Figure 8B). We only observed 64 genes that were differentially expressed in livers from CO-treated *Tiparp^H532A^* compared with *Tiparp^+/+^* mice (Supplementary Table 2). However in response to TCDD treatment, we identified 4542 genes with an absolute linear fold change of ≥2.0 (adjusted *p* value < .05) in *Tiparp^H532A^* mice (Supplementary Table 3) compared with 647 genes in *Tiparp^+/+^* mice (Supplementary Table 4), with 558 (86%) genes common to both datasets (Supplementary Table 6 and Figure 8C). In agreement with the increased liver fibrosis in TCDD-treated *Tiparp^H532A^* mice, we observed higher gene expression levels of transforming growth factor β (*Tgfb*), zinc finger E-box binding homeobox 2 (*Zeb2*) and collagen type I alpha 1 chain (*Col1a1*), all of which are associated with fibrosis. We identified 3867 genes that were significantly changed in *Tiparp^H532A^* mice when normalized against TCDD-treated *Tiparp^+/+^* samples. An overview of the number of differentially expressed genes in common between genotypes or treatment group contrasts illustrated the increased number of gene changes in TCDD *Tiparp^H532A^* mice (Figure 8D). The analysis also revealed that there were slightly more upregulated than downregulated genes among the different comparisons, with the exception of the TCDD-treated *Tiparp^H532A^* versus *Tiparp^+/+^* samples (Figure 8D). The top 10 upregulated and downregulated genes for the different contrasts are shown in Figure 8E. Of the 64 genes that were differentially expressed in CO-treated animals, the top 4 upregulated genes included serum amyloid A2 (*Saa2*), *Saa1*, lipocalin 2 (*Lcn2*), and metallothionein (*Mt1*), which are associated with inflammation and stress responses (Figure 8E). The top upregulated genes in the TCDD- vs CO-treated *Tiparp^H532A^* mice and TCDD- versus CO-treated *Tiparp^+/+^* mice included well-known AHR gene battery transcripts, whereas the top downregulated genes differed between the contrasts. The contrast of TCDD-treated *Tiparp^H532A^* versus similarly treated *Tiparp^+/+^* reflected the top differentially expressed hepatic genes in *Tiparp^H532A^* mice and confirmed the repression of *Cyp7a1* (Figure 7I). Ingenuity pathway analysis (IPA) was used to home in on genes in the AHR pathway among the different treatment and genotype contrasts (Supplementary Table 6). The analysis revealed that most, but not all, of the genes were significantly changed in TCDD-treated *Tiparp^H532A^* compared with CO or TCDD-treated *Tiparp^+/+^* versus TCDD-treated *Tiparp^+/+^* compared with CO contrasts. A partial list is provided in Figure 9A. A comparison of the hepatic gene expression changes with published AHR ChIP-seq in 2 h TCDD-treated liver samples (Dere *et al.*, 2011) revealed that 1549 (34.1%) and 204 (31.5%) genes overlapped with AHR bound genes (Figure 9B). We used IPA to identify the top differentially regulated pathways among the different treatment and genotype contrasts (Supplementary Table 7). A selection of some of the top pathways revealed that leukotriene biosynthesis was upregulated in TCDD-treated *Tiparp^H532A^*, but unchanged in TCDD-treated *Tiparp^+/+^* (Figure 9C). In contrast, nicotine and melatonin degradation pathways were downregulated in TCDD-treated *Tiparp^H532A^* but upregulated TCDD-treated *Tiparp^+/+^* mice. The majority of the pathways identified in TCDD-treated *Tiparp^H532A^* were downregulated, including NAD^+^ biosynthesis and tryptophan degradation. No differentially regulated pathways were identified in CO-treated *Tiparp^H532A^* compared with *Tiparp^+/+^* samples. This analysis supports the increased TCDD responsiveness of *Tiparp^H532A^* mice and shows that loss of TIPARP catalytic activity increases the susceptibility of mice to TCDD toxicity.

#### Pharmacologic Inhibition of TIPARP Increases TCDD-induced AHR-Regulated Target Gene Levels

**Figure 10.**
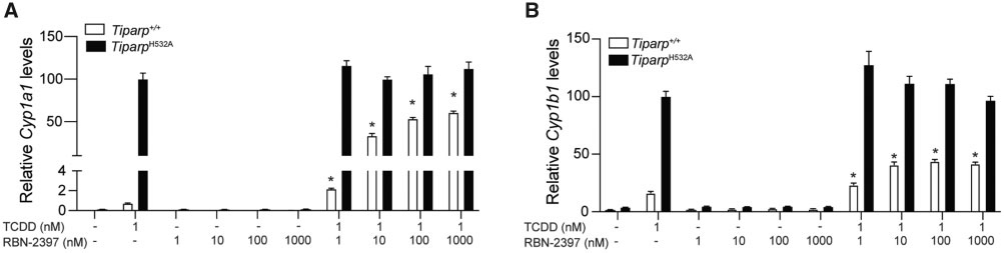
Pharmacological inhibition of 2,3,7,8-tetrachlorodibenzo-*p*-dioxin (TCDD)-inducible poly- adenosine diphosphate-ribose polymerase (TIPARP) increases aryl hydrocarbon receptor (AHR) signaling in mouse embryonic fibroblasts (MEFs) isolated from *Tiparp^+/+^* but not *Tiparp^H532A^* mice. Increased TCDD-induced mRNA expression levels of (A) *Cyp1a1*, and (B) *Cyp1b1* in MEFs isolated from *Tiparp^H532A^* and *Tiparp^+/+^* mice treated with TCDD alone or in combination with increasing concentrations of RBN-2397. Real time-quantitative PCR data are shown relative to the gene-specific expression level observed for *Tiparp^H532A^* MEFs exposed to 1 nM TCDD. *p <* *.05 by 1-way analysis of variance followed by Tukey’s post hoc test compared with genotype-matched TCDD-treated MEFs. Figure depicts the mean ± SEM (*n *=* *3).

Ribon Therapeutics recently presented a potent and selective small molecule inhibitor of TIPARP (PARP7), referred to as RBN-2397 that enhances IFN-I signaling and causes lung cancer regression in xenograft models (Vasbinder et al., 2020). RBN-2397 is currently in a phase I clinical trial to assess its potential therapeutic benefit to patients with advanced-stage solid tumors (NCT04053673). We investigated the effect of RBN-2397 on TCDD-induced *Cyp1a1* and *Cyp1b1* transcript levels in *Tiparp^+/+^* and *Tiparp^H532A^* MEFs (Figs. 10A and 10B). No significant increases in *Cyp1a1* and *Cyp1b1* levels were observed with RBN-2397 treatment alone. Cotreatment of 1 nM TCDD with increasing concentrations of RBN-2397 resulted in a concentration-dependent increase in both *Cyp1a1* and *Cyp1b1* levels in *Tiparp^+/+^* MEFs. RBN-2397 had no effect on TCDD-induced *Cyp1a1* and *Cyp1b1* mRNA levels in *Tiparp^H532A^* MEFs. These data indicate that pharmacological inhibition of TIPARP increases AHR signaling and may impact AHR ligand toxicity.

## DISCUSSION

Here, we describe the generation of *Tiparp^H532A^* catalytic mutant mice and show that loss of TIPARP’s mono-ADP-ribosyltransferase activity is sufficient to increase sensitivity to TCDD-induced toxicity. We show that a single injection of a normally nonlethal dose of TCDD to *Tiparp^H532A^* induces a lethal wasting syndrome characterized by increased AHR signaling, steatohepatitis, liver fibrosis, and loss of WAT. These findings provide further support for the importance of TIPARP in AHR signaling and for its role in protecting against TCDD-induced toxicity (Ahmed *et al.*, 2015; Matthews, 2017). They also show that TIPARP’s protective role requires its catalytic activity, suggesting that pharmacological inhibition of TIPARP could enhance TCDD-mediated toxicities.

Unlike whole-body *Tiparp*^−^^/^^−^ and hepatocyte-specific *Tiparp*^−/−^ (*Tiparp^Hep^*^−/−^*)*, TIPARP mRNA levels and protein levels increased in response to TCDD in *Tiparp^H532A^* mice. This was expected since TIPARP is an AHR target gene; however, the lack of catalytic activity prevents its ability to function as a repressor of AHR. TIPARP is an unstable protein that is rapidly degraded (Kamata *et al.*, 2021), but the introduction of the H532A mutation into endogenous TIPARP increased its stability. We have observed similar findings with pharmacological inhibition of TIPARP (Rodriguez *et al.*, 2021), suggesting that TIPARP’s catalytic activity regulates its protein stability. HIF1α and androgen receptor (AR) also regulate TIPARP protein levels. HIF1α increases and AR activation prevents TIPARP degradation, but whether AR affects TIPARP catalytic activity is not known (Cheng *et al.*, 2019; Kamata *et al.*, 2021).

*Tiparp*^−/−^ and *Tiparp^Hep^*^−/−^ mice exhibit increased AHR activity and increased sensitivity to TCDD-induced toxicities and wasting syndrome (Ahmed *et al.*, 2015; Hutin *et al.*, 2018). This increased sensitivity to AHR ligands is not limited to TCDD, since 3-methylcholanthrene treatment also causes severe toxicity and lethality to *Tiparp*^−/−^ mice (Cho *et al.*, 2019). Overall, the TCDD-treated *Tiparp^H532A^* mice exhibited many, if not all, of the same phenotypes as similarly treated *Tiparp*^−/−^ and *Tiparp^Hep^*^−/−^ mice. Common among TCDD-treated *Tiparp^H532A^*, *Tiparp*^−/−^, and *Tiparp^Hep^*^−/−^ mice is the loss of body weight and WAT as well as death occurring 5–10 days after a single i.p. injection of 10 µg/kg TCDD (Ahmed *et al.*, 2015; Hutin *et al.*, 2018). TCDD induces body weight and adipose tissue loss in many species; however, pair-feeding studies and total parenteral nutrition failed to identify the cause of the body weight and adipose tissue loss (Linden *et al.*, 2010). One possibility is that the extensive hepatotoxicity, steatohepatitis and fibrosis in TCDD-treated *Tiparp^H532A^* mice impairs liver homeostasis and consequently alters intestinal nutrient absorption (Kalaitzakis, 2014). Similar to previous studies, TCDD-treated *Tiparp^H532A^* mice display a number of alterations in lipid homeostasis (Ahmed *et al.*, 2015; Boverhof *et al.*, 2006; Duval *et al.*, 2017; Hutin *et al.*, 2018), including increased expression of genes regulating lipid uptake, but decreased expression of those involved in *de novo* lipogenesis and fatty acid β-oxidation. Hepatic gene expression profiling revealed approximately 7-fold more genes that were significantly differentially expressed in TCDD-treated *Tiparp^H532A^* compared with *Tiparp^+/+^* mice. However, *Cyp1a1* mRNA was not significantly different 6 days after TCDD treatment. This was in contrast to *Cyp1b1*, *Nqo1*, *Tiparp*, *Ahrr*, and *Fgf21* mRNA levels. These differences could be due to differential temporal regulation of these genes compared with *Cyp1a1*, differences in RNA stability, and/or that some of these genes might be regulated in response to TCDD-induced hepatotoxicity resulting in the induction or activation of other transcription factors that contribute to their regulation. For example, *Cyp1b1*, *Tiparp*, and *Ahrr* are regulated by multiple transcription factors, stress response, inflammation, and other signal transduction pathways (Brandstatter *et al.*, 2016; Falero-Perez *et al.*, 2018; Lano *et al.*, 2020; Matthews, 2017). *NqoI* is also regulated by Nuclear factor erythroid 2-related factor 2 (NFE2l2; NRF2) in response to oxidative stress (Nioi *et al.*, 2003), which would be expected given the extensive hepatotoxicity observed in TCDD-treated *Tiparp^H532A^* mice. Hepatic *Fgf21* expression is increased in the patients with hepatic steatosis, in mouse models of obesity or nonalcoholic fatty liver disease, and in response to ER stress (Tezze *et al.*, 2019). Moreover, the reduced hepatic AHR protein levels observed in *Tiparp^H532A^* mice 6 days after TCDD treatment may also contribute to the lack of a significant increase in *Cyp1a1* mRNA levels compared with the other AHR-regulated genes examined. Nonetheless, approximately one-third of the identified genes overlapped with AHR bound regions, supporting overall increased AHR signaling in *Tiparp^H532A^* mice. In addition, the lower mRNA levels of *Pck1* and *Cyp7a1* suggest altered glucose and bile acid homeostasis via decreased cholesterol catabolism, which support increased use of lipids for energy and possibly reduced lipid absorption resulting from altered bile acid homeostasis (Fader *et al.*, 2017). Similar findings were observed in Long Evans rats, where expression levels of *Cyp7a1* were reduced to 1% of control animals 4 days after treatment with 50 µg/kg TCDD (Linden *et al.*, 2014). We saw repression of *Cyp7a1* mRNA levels only in TCDD-treated *Tiparp^H532A^* mice that showed severe wasting, but it was increased in similarly treated *Tiparp^+/+^* mice. In addition, lower levels of 3-hydroxy-3-methylglutaryl-CoA synthase 2 (*Hmgcs2*), the rate limiting step in ketogenesis, were only observed in TCDD-treated *Tiparp^H532A^* mice. Lower levels of HMGCS2 would reduce ketone body formation and thus an important potential energy source. Combined with the lower levels of *Cyp7a1*, this could contribute to increased liver toxicity through an accumulation of cholesterol and or bile acid toxicity (Ahmed *et al.*, 2015; van der Geest *et al.*, 2016).

The increased levels of the fasting-adaptation hormone and AHR target gene *Fgf21* (Fazeli *et al.*, 2015), which affects glucose and lipid metabolism, may also be an important contributing factor to the metabolic toxicity observed in TCDD-treated *Tiparp^H532A^* mice. Studies in TCDD-treated Long Evans rats provide evidence that the endocrine system responds by trying to restore energy homeostasis (Linden *et al.*, 2014). They observed increased *Fgf21* and lower *Pck1* transcript levels, which counteracts hypoglycemia and energy imbalance. However, these signals appear to be incorrectly interpreted in the CNS and wasting syndrome persists. These data support a significant role for AHR in energy homeostasis, but given that the magnitude of TCDD-induced wasting varies among laboratory animals, the role of AHR in energy homeostasis might also vary among species.

Several cellular pathways were altered in TCDD-treated *Tiparp^H532A^* compared with *Tiparp^+/+^* mice, including xenobiotic metabolism, constitutive androstane receptor, neuroinflammation, and leukotriene biosynthesis. The latter was upregulated in treated *Tiparp^H532A^* but unchanged in *Tiparp^+/+^*, and may contribute to the elevated inflammation in *Tiparp^H532A^* mice. Many other pathways were downregulated in *Tiparp^H532A^*, but unaffected in *Tiparp^+/+^*, including tryptophan degradation and NAD^+^ biosynthesis. Reduced tryptophan degradation could reduce the levels of some endogenous AHR ligands, including KYN and kynurenic acid. An altered KYN/tryptophan ratio is linked to various pathological conditions, immune activation, and disease states. Reduced tryptophan degradation may also contribute to reduced *de novo* NAD^+^ levels. NAD^+^ levels are also reduced in *Tiparp*^−/−^ and *Tiparp^Hep^*^−/−^ mice (Hutin *et al.*, 2018). NAD^+^ repletion has been shown to prevent TCDD-induced thymic atrophy and hepatosteatosis in chicken embryos (Diani-Moore *et al.*, 2017). Thus, the reduced NAD^+^ biosynthesis could compound the TCDD-induced toxicity responses in *Tiparp^H532A^* mice.

Here, we show that the loss of TIPARP catalytic activity, and not just its protein expression, increases sensitivity to TCDD toxicity. Thus, identifying protein substrates of TIPARP, and the consequences of ADP-ribosylation on their function will be important future studies that can be combined with gene expression profiling to fully understand how TIPARP protects against TCDD toxicity. AHR is a TIPARP substrate, but whether loss of AHR ADP-ribosylation is enough to increase sensitivity to TCDD or if there are other proteins involved is not known. Recently, chemical genetics using targeted TIPARP mutation and NAD^+^ analogs have been used to characterize the TIPARP (PARP7) ADP-ribosylated proteome in HEK293, ovarian (OVCAR4) cancer cells and HeLa cells (Palavalli Parsons *et al.*, 2021; Rodriguez *et al.*, 2021). Although metabolic targets were found among the TIPARP substrates, including the AHR target gene products ELAVL1 and SREBP1, many of the identified substrates were involved in inflammation, cell-cell adhesion and cytoskeletal signaling. However, the most significant upregulated cellular pathways were ribosomes, RNA transport and DNA repair. The impact of TIPARP ADP-ribosylation on protein function is incompletely understood, but current data suggest that TIPARP regulates protein stability through increased ubiquitination (Palavalli Parsons *et al.*, 2021; Rodriguez *et al.*, 2021). Characterizing the TCDD-induced TIPARP ADP-ribosylated proteome will be important to fully understand the increased sensitivity of *Tiparp^H532A^* mice, and may also provide new insight into TCDD-induced toxicity and wasting syndrome.

TIPARP has recently emerged as a potential therapeutic target for cancer treatment. TIPARP has both cancer intrinsic effects and extrinsic effects via its inhibitory role in type I interferon (IFN) signaling, and because of this it has emerged as a potential therapeutic target for cancer treatment. TIPARP knockdown reduces ovarian cancer cell growth and motility partly through the ADP-ribosylation of α-tubulin (Palavalli Parsons *et al.*, 2021). Type I IFNs regulate tumor infiltrating immune cells and are required for an effective immune response against cancer cells, but also reduce tumor cell growth inhibition and increase apoptosis (Musella *et al.*, 2017). Since TIPARP reduces type I IFN signaling, inhibition or loss of TIPARP expression would prevent tumors from evading the immune system, resulting in increased anti-tumorigenic responses. Ribon Therapeutics has recently described RBN-2397, a TIPARP-specific inhibitor that is currently in a phase 1 clinical trial designed to assess its antitumor activity in patients with advanced-stage solid tumors (NCT04053673). RBN-2397-mediated TIPARP inhibition prevents CT26 colon tumor growth in a type I IFN signaling dependent manner (Vasbinder et al., 2020). Conversely, TIPARP knockdown promotes tumor growth in an MCF-7 xenograft model (Zhang *et al.*, 2020). This implies that TIPARP’s effects on cancer may be context and cancer type specific. Moreover, loss of TIPARP catalytic activity in both TCDD-induced and natural ligand mediated regulation of *Cyp1a1* and *Cyp1b1* was enhanced by cotreatment with RBN-2397. Based on the studies presented here, it will be important to test how RBN-2397 or other TIPARP inhibitors affect AHR biology and TCDD-mediated toxicity.

In summary, we provide evidence that TIPARP catalytic activity and ADP-ribosylation is involved in regulating TCDD toxicity, and potentially in regulating the biological actions of AHR following its activation by endogenous or dietary ligands. Our *Tiparp^H532A^* mutant mice provide a unique opportunity to study TIPARP actions that are dependent on its catalytic activity and not just due to loss of TIPARP protein. These mice will also be instrumental in characterizing the specificity and therapeutic potential of TIPARP inhibitors. 

## SUPPLEMENTARY DATA

Supplementary data are available at *Toxicological Sciences* online.

## FUNDING

Canadian Institutes of Health Research (CIHR) operating grants (MOP-494265 and MOP-125919) and the Johan Throne Holst Foundation to J.M. A.S.L. is a recipient of a Natural Sciences and Engineering Research Council (NSERC) of Canada postdoctoral fellowship. 

## DECLARATION OF CONFLICTING INTERESTS

The authors declared no potential conflicts of interest with respect to the research, authorship, and/or publication of this article. 

## Supplementary Material

kfab075_Supplementary_Data
