# Supplementary material for: 2,3,7,8-Tetrachlorodibenzo-p-Dioxin (TCDD)-Inducible Poly-ADP-Ribose Polymerase (TIPARP/PARP7) Catalytic Mutant Mice (TiparpH532A) Exhibit Increased Sensitivity to TCDD-Induced Hepatotoxicity and Lethality
Source: Toxicol Sci. 2021 Jun 15;183(1):154–69. doi: 10.1093/toxsci/kfab075 (PMC8404992; doi:10.1093/toxsci/kfab075)
Supplement: kfab075_Supplementary_Data [file kfab075_supplementary_data.zip › toxsci-21-0155-File003.pdf]

Supplementary Data Description

There are 7 supplementary tables. Table S1 lists the qPCR primers used for gene expression analyses and genotyping. Tables S2-S5 include gene lists and analyses for different treatment and genotype comparisons that were determined using RNA-sequencing. Tables S6 and S7 summarize changes in AHR gene battery genes and pathway analysis of the RNA-sequencing data that was done using ingenuity pathway analysis.
